# Prognostic value of systemic inflammation response index in nasopharyngeal carcinoma with negative Epstein-Barr virus DNA

**DOI:** 10.1186/s12885-022-09942-1

**Published:** 2022-08-05

**Authors:** Xiaofei Yuan, Hua Yang, Fangfang Zeng, Shiyu Zhou, Shuting Wu, Yue Yuan, Linchong Cui, Huiru Feng, Danfan Lin, Zilu Chen, Xiong Liu, Jing Chen, Fan Wang

**Affiliations:** 1 Department of Otolaryngology-Head and Neck Surgery, Nanfang Hospital, Southern Medical University, Baiyun District, Jingxi Street, Guangzhou, 510515 People’s Republic of China; 2 Department of Biostatistics, School of Public Health, (Guangdong Provincial Key Laboratory of Tropical Disease Research), Southern Medical University, Guangzhou, 510515 Guangdong China; 3 School of Traditional Chinese Medicine, Southern Medical University, Guangzhou, 510515 Guangdong China

**Keywords:** Nasopharyngeal carcinoma, Systemic inflammation response index, Epstein–Barr virus, Prognosis, Risk stratification

## Abstract

**Background:**

Inflammatory parameters and Epstein–Barr virus (EBV) DNA status have been confirmed to be associated with prognosis in nasopharyngeal carcinoma (NPC) patients. However, there are few in-depth studies on the prognosis of NPC patients with negative EBV DNA. Our study aimed to look for inflammatory biomarkers that can identify disease progression in NPC patients with negative EBV DNA.

**Methods:**

A total of 795 NPC patients were recruited, and ultimately 325 NPC patients with negative EBV DNA were included in this study (170 in training cohort and 155 in validation cohort). Kaplan–Meier method and log-rank test were used to analyze progression-free survival (PFS) and overall survival (OS). The multivariate analysis of Cox proportional hazards regression model was used to determine the independent prognostic factors. Receiver operating characteristic (ROC) curves were used to assess prognostic value. The logistic regression was used to evaluate the relationship between EBV DNA status and inflammatory parameters. The correlation between clinical characteristics was analyzed by the chi-squared test or the Fisher’s exact test.

**Results:**

The optimal cutoff point for the SIRI was 1.12. The EBV DNA-negative NPC patients with high SIRI level had worse PFS and OS (all *p* < 0.001). In multivariate Cox proportional hazard models analysis, SIRI was an independent prognostic factor for PFS and OS (all *p* < 0.05), and had higher prognostic value than other indicators. Above results were found in the training cohort and confirmed in the validation cohort. In addition, EBV DNA status was not associated with any inflammatory parameters.

**Conclusions:**

The SIRI can provide more accurate risk stratification and better prognostic prediction for NPC patients with negative EBV DNA.

**Supplementary Information:**

The online version contains supplementary material available at 10.1186/s12885-022-09942-1.

## Introduction

Nasopharyngeal carcinoma (NPC) is an epithelial carcinoma originating from the nasopharyngeal mucosa that is apparently different from other head and neck cancers [1]. NPC has extremely unbalanced geographical global distributions and is prevalent in Southeast Asia, especially in Southern China [2]. Since NPC is sensitive to chemoradiation, the main treatment for NPC is intensity-modulated radiation therapy (IMRT) combined with or without chemotherapy [3]. However, approximately 30% of patients still have a poor survival rate due to local recurrence and distant metastasis [3, 4]. Risk stratification through reliable predictors is necessary for individualized treatment which has important clinical value for better prognosis and improved survival.

Epstein–Barr virus (EBV) infection has been regarded as one of the risk factors contributing to the development of NPC [5]. The detection of plasma EBV DNA has been proven to be a reliable biomarker for population screening, prognosis, precise treatment and posttreatment monitoring of NPC patients [6–9]. High plasma EBV DNA load can reflect advanced TNM classification, tumor burden and residual disease of NPC patients [10–12]. Many previous studies have demonstrated that plasma EBV DNA is closely related to the prognosis of NPC patients [7, 13, 14]. About 40% of NPC patients are negative for EBV quantification before treatment. The prognosis of EBV DNA-positive patients is much worse than that of EBV DNA-negative patients. However, some NPC patients with negative EBV DNA still have poor survival. According to our previous study [15], the 5-year PFS and OS of NPC patients with low EBV DNA level were approximately 80% and 90%, respectively. Therefore, looking for biomarkers that can identify disease progression in NPC patients with negative EBV DNA may bring prognosis benefits for these patients.

Systemic inflammation is one of the basic components of the tumor microenvironment and can affect the pathogenesis of cancer patients [16]. Inflammation and immune surveillance have been recently established as cancer hallmarks, showing an essential role in the progression, recurrence and metastasis of cancer [1]. In recent years, immune-inflammation indexes including the systemic immune-inflammation index (SII) based on three leukocytes (peripheral blood neutrophils, platelets and lymphocytes) and the systemic inflammation response index (SIRI) based on three leukocytes (peripheral blood neutrophils, monocytes and lymphocytes) were investigated in various cancers. In the study by Chen et al. [17], high SII levels were associated with worse overall survival (OS) and disease-free survival (DFS) in colorectal cancer. A previous study was followed by Zeng et al. [18], which reported that pretreatment SII in NPC patients were significantly higher than those in patients with chronic rhinitis, and could be used as prognostic indicators in NPC patients. A retrospective study involving 177 patients with pancreatic cancer after chemotherapy in a training cohort, showed that a high pretreatment SIRI was significantly related to poor time to progression (TTP) and OS, which was verified in the validation cohort [19]. Chen et al. [20] first demonstrated that SIRI was an important prognostic biomarker for patients with NPC in 2018, and an increase in the SIRI was associated with worse OS. As mentioned earlier, some NPC patients with negative EBV DNA also have recurrence and metastasis. In view of the important role of the inflammatory indicators in cancer prognosis, we tried to apply these indicators to NPC patients with negative EBV DNA to observe whether they can effectively distinguish high-risk patients.

Therefore, we conducted this retrospective study to evaluate the prognostic value of inflammatory parameters in NPC patients with negative EBV DNA and to validate all results. This study aimed to strengthen the survival prediction, risk stratification and precise treatment of such patients.

## Materials and methods

### Case selection

Between January 2005 and December 2015, we retrospectively recruited 795 patients who were diagnosed with NPC at Nanfang Hospital of Southern Medical University. All patients were confirmed by pathological examinations and staged according to the eighth edition of the American Joint Committee on Cancer (AJCC) staging system. This retrospective study was approved by the Ethics Committee of Nanfang Hospital of Southern Medical University (Ethical review approval no.: NFEC-2017–165).

### Inclusion and exclusion criteria

The inclusion criteria in this study were as follows: (a) patients with NPC confirmed by histopathology; (b) patient with complete medical treatment time records and history records; (c) patients with at least one complete record of a peripheral blood count before treatment; and (d) patients with at least one EBV DNA test before treatment. The exclusion criteria were as follows: (a) patients with prior malignancy; (b) patients with a history of previous anticancer therapy; (c) patients with non-WHO pathological types; (d) patients with uncontrolled infection; or (e) patients with positive EBV DNA.

Finally, a total of 325 NPC patients with negative EBV DNA were included. All these patients were divided into the training cohort and validation cohort by random number.

### Hematological examination

The peripheral blood cells of all patients were collected and tested for leukocyte, neutrophil, lymphocyte, monocyte, and platelet counts within 1 week before therapy. The measurements of plasma EBV DNA were performed within 1 month before therapy. The SII was defined as (neutrophil*platelet)/lymphocyte; the SIRI is defined as (neutrophil*monocyte)/lymphocyte. All peripheral blood cell and EBV DNA assessments were performed in the Laboratory Medicine Center of Nanfang Hospital, Southern Medical University according to standard operating procedures. The cutoff level chosen to classify the patients into the negative and positive EBV DNA groups was 500 copies/mL before treatment, referring to the threshold of Laboratory Medicine Center, Nanfang Hospital, Southern Medical University, in this study (The detection method for EBV DNA in Supplementary file 1.).

### Treatment with guideline

All patients developed treatment plans according to the then latest NCCN (National Comprehensive Cancer Network) guidelines. The general principles of treatments were as follows: all patients received IMRT, patients with stage I received IMRT alone, patients with stage II received concurrent chemoradiotherapy, patients with stage III/IV received concurrent chemoradiotherapy, induction chemotherapy, and/or adjuvant chemotherapy.

The general principles of radiation therapy were as follows: all patients were treated with 2.12–2.24 Gy each time, 5 times a week, with IMRT for 6–8 weeks. The total prescribed IMRT doses were 70–74 Gy to the gross tumor volume of the nasopharynx (GTVnx), 66–70 Gy to the positive neck lymph node area (GTVnd), 60–62 Gy to the high-risk sites defined as clinical target volume (CTV1), and 50–56 Gy to the low-risk sites defined as clinical target volume (CTV2).

The principles of chemotherapy were as follows: induction chemotherapy (IC) (1–2 cycles) and adjuvant chemotherapy (AC) (1–4 cycles) included TP, TPF, and PF. The TP regimen was paclitaxel 135 mg/m2/d or docetaxel 60 mg/m2/d on day 1 and cisplatin 25 mg/m2/d on days 1 to 3, the TPF regimen was paclitaxel 135 mg/m2/d or docetaxel 60 mg/m2/d on day 1, cisplatin 25 mg/m2/d on days 1 to 3, and 5-fluorouracil 600 mg/m2/d on days 1 to 5, and the PF regimen was cisplatin 25 mg/m2/d on days 1 to 3 and 5-fluorouracil 600 mg/m2/d on days 1 to 5. The concurrent chemoradiotherapy (CCRT) (1–2 cycles) included cisplatin monotherapy and TP. The cisplatin monotherapy regimen was cisplatin 25 mg/m2/d on days 1 to 3, and the TP regimen was paclitaxel 135 mg/m2/d or docetaxel 60 mg/m^2^/d on day 1 and cisplatin 25 mg/m2/d on days 1 to 3. All chemotherapy regimens were administered every 21 days as a complete cycle.

### Follow-up and endpoint

The follow-up schedule was conducted strictly in accordance with the following 3, 6 and 12 months in the first year after therapy, every 6 months in the second and third years, and once a year thereafter. Each follow-up evaluation included head and neck physical examination, abdominal ultrasound, nasopharyngeal endoscopy, chest radiograph, peripheral blood examination, whole-body positron emission tomography-computed tomography (PET-CT) or emission computed tomography (ECT), and nasopharyngeal and neck magnetic resonance imaging (MRI). For cases with suspected recurrence of nasopharyngeal neck tumor or distant metastasis of cervical lymph nodes, biopsy or needle biopsy was performed on the suspected site to make a definitive diagnosis. The primary outcomes of this study were progression-free survival (PFS) and overall survival (OS). PFS was defined as the time from the initial pathological diagnosis of NPC to the date of disease progression or death from any cause. OS was defined as the time between the initial pathological diagnosis of NPC and all-cause death, or at the last follow-up.

### Statistical analysis

The optimal cutoff values of the SII and SIRI were determined using X-tile 3.6.1 software (Robert L Camp, Yale University, New Haven, CT, USA) [21, 22]. The cutoff values were plotted by X-tile 3.6.1 software, and other figures were plotted by GraphPad Prism V8.0. The chi-squared test and the Fisher’s exact test were used to explore the relationship between clinical characteristics and different variables. Logistic regression was used to estimate the odds ratio (OR) and 95%CI to evaluate the association between inflammatory parameters and EBV DNA status (negative vs. positive). Survival curves were analyzed using the Kaplan–Meier method and compared using the log-rank test. Univariate and multivariate Cox proportional hazards regressions were conducted to evaluate the prognostic significance of each variable with respect to PFS and OS. ROC curve analysis was performed to compare the different prognostic values. All variables reaching statistical significance in univariate analysis were included in multivariate Cox proportional hazards regression analysis. A two-tailed *p* value of less than 0.05 was considered statistically significant. All results were validated by the validation cohort. All statistical analyses were performed using the SPSS (Statistical Packages for Social Science) version 23.0 (IBM, Corporation).

## Results

### The optimal cutoff values of the SII, SIRI and the peripheral blood cells

**Fig. 1:**
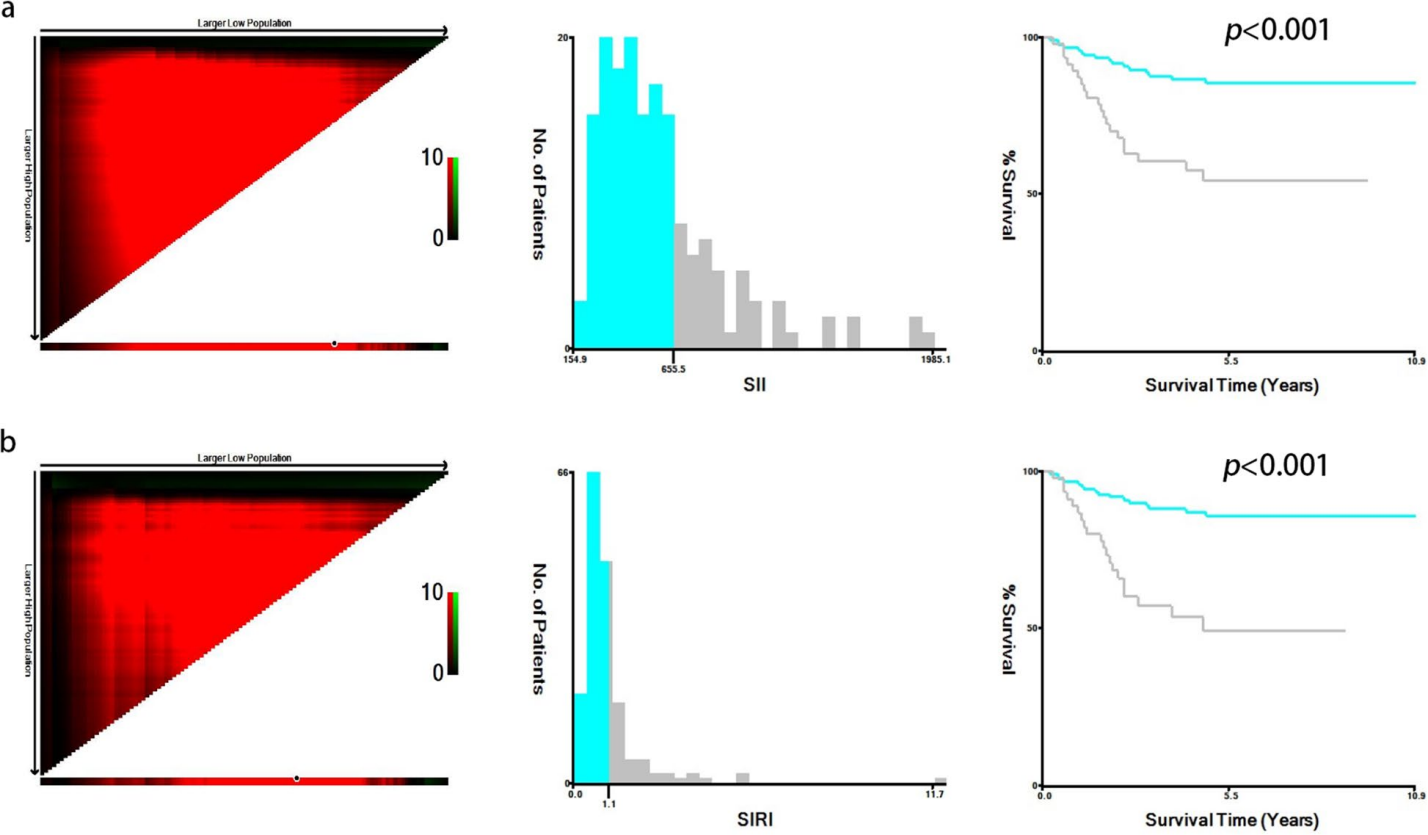
X-tile analysis of survival data of NPC patients. **a** The optimal cut-off value for the SII was 655.54 (chi square = 18.922, *p* < 0.001); **b** The optimal cut-off value for the SIRI was 1.12 (chi square = 23.425, *p* < 0.001)

The X-tile 3.6.1 software was used to evaluate the optimal cutoff values of the SII and SIRI for progression outcome in training cohort. The analysis demonstrated that the optimal cutoff values of the SII and SIRI were 655.54 (*p* < 0.001) and 1.12 (*p* < 0.001), respectively (Fig. 1). The optimal cutoff values of other peripheral blood cells were determined as the upper limits of the normal values as defined by the Laboratory Medicine Center of Nanfang Hospital, Southern Medical University (leukocytes: 9.5*10^9^/L; neutrophil: 6.3*10^9^/L; lymphocyte: 3.2*10^9^/L; monocyte: 0.6*10^9^/L; platelet: 300*10^9^/L).

### Patients characteristics and follow-up

The clinical characteristics of the 170 NPC patients in training cohort and 155 NPC patients in validation cohort were listed in Table 1. In training cohort, 112 (65.9%) were male and 58 (34.1%) were female, the median age was 47 years old, the median follow-up duration was 62.0 months. During the long-term follow-up, 39 (22.9%) patients experienced disease progression, 23 (13.5%) patients experienced locoregional relapse, 14 (8.2%) patients experienced distant metastasis and 20 (11.8%) patients died. The 5-year PFS and OS rates were 78.8% and 89.4%, respectively. In validation cohort, 117 (75.5%) were male and 38 (24.5%) were female, the median age was 46 years old, the median follow-up duration was 60.0 months. During the long-term follow-up, 31 (20.0%) patients experienced disease progression, 10 (6.5%) patients experienced locoregional relapse, 21 (13.5%) patients experienced distant metastasis and 20 (12.9%) patients died. The 5-year PFS and OS rates were 80.6% and 87.7%, respectively.

**Table 1:** Baseline characteristics of NPC patients with negative EBV DNA (*n* = 325)

Variables	Training cohort (*n* = 170)	Validation cohort (*n* = 155)
	No.(%)	No.(%)
Gender		
male	112 (65.9)	117 (75.5)
female	58 (34.1)	38 (24.5)
Age		
≤ 55	134 (78.8)	126 (81.3)
> 55	36 (21.2)	29 (18.7)
Smoke		
no	113 (66.5)	89 (57.4)
yes	57 (33.5)	66 (42.6)
AJCC stage (8th)		
I	14 (8.2)	23 (14.8)
II	37 (21.8)	25 (16.1)
III	44 (25.9)	50 (32.3)
IVa	69 (40.6)	55 (35.5)
IVb	6 (3.5)	2 (1.3)
Tumor classification		
T1	36 (21.2)	48 (31.0)
T2	37 (21.8)	36 (23.2)
T3	29 (17.1)	26 (16.8)
T4	68 (39.9)	45 (29.0)
Node classification		
N0	30 (17.6)	39 (25.2)
N1	59 (34.7)	40 (25.8)
N2	75 (44.1)	63 (40.6)
N3	6 (3.5)	13 (8.4)
Metastasis		
Non-metastasis	164 (96.5)	153 (98.7)
Metastasis	6 (3.5)	2 (1.3)
WHO pathologic type		
TypeI	0 (0)	1 (0.6)
TypeII	15 (8.8)	17 (11.0)
TypeIII	155 (91.2)	137 (88.4)
Leukocytes		
≤ 9.5	159 (93.5)	140 (90.3)
> 9.5	11 (6.5)	15 (9.7)
Neutrophils		
≤ 6.3	157 (92.4)	138 (94.8)
> 6.3	13 (7.6)	17 (11.0)
Lymphocytes		
≤ 3.2	164 (96.5)	147 (94.8)
> 3.2	6 (3.5)	8 (5.2)
Monocytes		
≤ 0.6	151 (88.8)	137 (88.4)
> 0.6	19 (11.2)	18 (11.6)
Platelets		
≤ 300	143 (84.1)	132 (85.2)
> 300	27 (15.9)	23 (14.8)
SII		
≤ 655.54	123 (72.4)	104 (67.2)
> 655.54	47 (27.6)	51 (32.8)
SIRI		
≤ 1.12	125 (73.5)	110 (71.0)
> 1.12	45 (26.5)	45 (29.0)

*EBV DNA* Epstein-Barr virus DNA, *AJCC* American Joint Committee on Cancer, *WHO* World Health Organization, *SII* Systemic immune-inflammation index, *SIRI* Systemic inflammation response index

### Prognostic significance of clinical features and inflammatory parameters

**Fig. 2:**
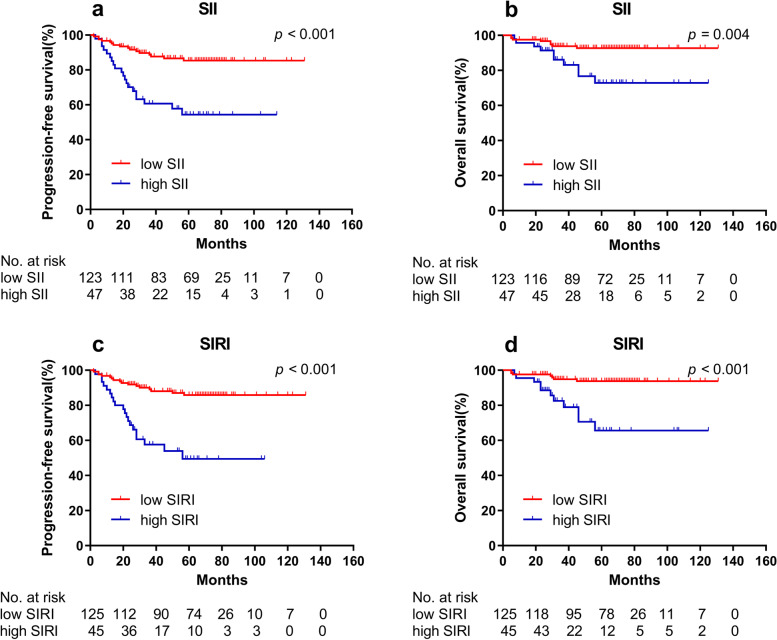
Kaplan–Meier curves for PFS and OS between different groups in training cohort. **a** Low SII group and high SII group for PFS; **b** Low SII group and high SII group for OS; **c** Low SIRI group and high SIRI group for PFS; **d** Low SIRI group and high SIRI group for O

**Fig. 3:**
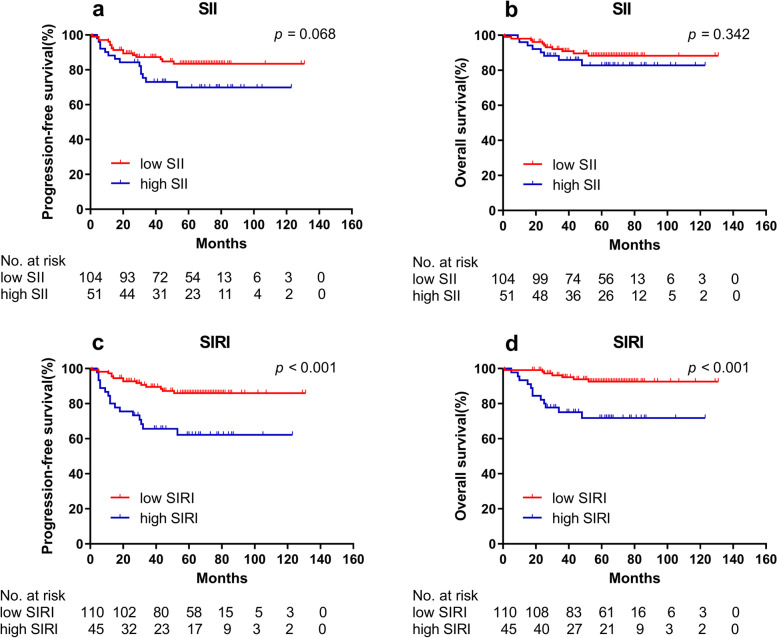
Kaplan–Meier curves for PFS and OS between different groups in validation cohort. **a** Low SII group and high SII group for PFS; **b** Low SII group and high SII group for OS; **c** Low SIRI group and high SIRI group for PFS; **d** Low SIRI group and high SIRI group for OS

The Kaplan–Meier survival curves for survival analyses showed that both higher SII group and higher SIRI group were significantly associated to worse PFS and worse OS in training cohort (all *p* < 0.01) (Fig. 2). In validation cohort, only the higher SIRI group was significantly associated to worse PFS (*p* < 0.001) and worse OS (*p* < 0.001) (Fig. 3).

In the univariate Cox regression model, platelets (*p* = 0.001), SII (*p* < 0.001) and SIRI (*p* < 0.001) were significantly associated with PFS in training cohort. The age (*p* = 0.038), SII (*p* = 0.007) and SIRI (*p* < 0.001) were significantly associated with OS in training cohort (Table 2). The age, leukocytes, neutrophils, monocytes and SIRI were significantly associated with PFS and OS in validation cohort (all *p* < 0.01) (Table 3). All variables reaching statistical significance in univariate analysis were included in multivariate Cox proportional hazards regression analysis.

**Table 2:** Univariate and Multivariate analysis of PFS and OS for NPC patients with negative EBV DNA in training cohort (*n* = 170)

Variables	Univariate analysis		Multivariate analysis	
	HR (95%CI)	*p*	HR (95%CI)	*p*
**PFS**				
Gender (female vs. male)	0.816 (0.401–1.658)	0.574	-	-
Age (> 55 vs. ≤ 55)	1.619 (0.780–3.362)	0.196	-	-
Smoke (yes vs. no)	1.121 (0.568–2.214)	0.741	-	-
AJCC stage (8th) (III-IVb vs. I-II)	1.602 (0.730–3.516)	0.240	-	-
Tumor classification (T_3-4_ vs. T_1-2_)	1.873 (0.921–3.809)	0.083	-	-
Node classification (N_2-3_ vs. N_0-1_)	1.021 (0.530–1.965)	0.951	-	-
Metastasis (Yes vs. No)	1.894 (0.454–7.893)	0.381	-	-
WHO pathologic type (TypeIIb vs. TypeIIa vs. TypeI)	1.154 (0.354–3.762)	0.813	-	-
Leukocytes (> 9.5 vs. ≤ 9.5)	1.835 (0.649–5.189)	0.253	-	-
Neutrophils (> 6.3 vs. ≤ 6.3)	2.133 (0.829–5.489)	0.116	-	-
Lymphocytes (> 3.2 vs. ≤ 3.2)	1.952 (0.468–8.145)	0.359	-	-
Monocytes (> 0.6 vs. ≤ 0.6)	1.355 (0.526–3.491)	0.529	-	-
Platelets (> 300 vs. ≤ 300)	3.248 (1.622–6.504)	**0.001**	1.976 (0.897–4.354)	0.091
SII (> 655.54 vs. ≤ 655.54)	3.876 (2.005–7.493)	** < 0.001**	1.467 (0.558–3.861)	0.437
SIRI (> 1.12 vs. ≤ 1.12)	4.471 (2.303–8.678)	** < 0.001**	3.032 (1.251–7.348)	**0.014**
**OS**				
Gender (female vs. male)	0.727 (0.259–2.038)	0.544	-	-
Age (> 55 vs. ≤ 55)	2.734 (1.058–7.065)	**0.038**	2.412 (0.928–6.271)	0.071
Smoke (yes vs. no)	2.010 (0.798–5.064)	0.139	-	-
AJCC stage (8th) (III-IVb vs. I-II)	3.763 (0.865–16.371)	0.077	-	-
Tumor classification (T_3-4_ vs. T_1-2_)	2.972 (0.978–9.035)	0.055	-	-
Node classification (N_2-3_ vs. N_0-1_)	1.135 (0.450–2.859)	0.789	-	-
Metastasis (Yes vs. No)	3.916 (0.899–17.064)	0.069	-	-
WHO pathologic type (TypeIIb vs. TypeIIa vs. TypeI)	1.814 (0.241–13.636)	0.563	-	-
Leukocytes (> 9.5 vs. ≤ 9.5)	Not estimable	-	Not estimable	-
Neutrophils (> 6.3 vs. ≤ 6.3)	Not estimable	-	Not estimable	-
Lymphocytes (> 3.2 vs. ≤ 3.2)	Not estimable	-	Not estimable	-
Monocytes (> 0.6 vs. ≤ 0.6)	0.525 (0.070–3.949)	0.531	-	-
Platelets (> 300 vs. ≤ 300)	2.282 (0.813–6.410)	0.117	-	-
SII (> 655.54 vs. ≤ 655.54)	3.563 (1.404–9.039)	**0.007**	1.416 (0.434–4.626)	0.564
SIRI (> 1.12 vs. ≤ 1.12)	5.527 (2.129–14.348)	** < 0.001**	4.141 (1.230–13.935)	**0.022**

*EBV DNA* Epstein-Barr virus DNA, *AJCC* American Joint Committee on Cancer, *WHO* World Health Organization, *SII* Systemic immune-inflammation index, *SIRI* Systemic inflammation response index

**Table 3:** Univariate and Multivariate analysis of PFS and OS for NPC patients with negative EBV DNA in validation cohort (*n* = 155)

Variables	Univariate analysis		Multivariate analysis	
	HR (95%CI)	*p*	HR (95%CI)	*p*
**PFS**				
Gender (female vs. male)	0.426 (0.149–1.221)	0.112	-	-
Age (> 55 vs. ≤ 55)	2.964 (1.410–6.232)	**0.004**	3.443 (1.561–7.594)	**0.002**
Smoke (yes vs. no)	1.480 (0.723–3.029)	0.283	-	-
AJCC stage (8th) (III-IVb vs. I-II)	2.587 (0.990–6.762)	0.053	-	-
Tumor classification (T_3-4_ vs. T_1-2_)	1.279 (0.625–2.618)	0.500	-	-
Node classification (N_2-3_ vs. N_0-1_)	1.738 (0.836–3.613)	0.139	-	-
Metastasis (Yes vs. No)	4.171 (0.567–30.672)	0.161	-	-
WHO pathologic type (TypeIIb vs. TypeIIa vs. TypeI)	0.557 (0.253–1.225)	0.146	-	-
Leukocytes (> 9.5 vs. ≤ 9.5)	3.502 (1.501–8.172)	**0.004**	1.851 (0.536–6.391)	0.330
Neutrophils (> 6.3 vs. ≤ 6.3)	3.718 (1.651–8.370)	**0.002**	1.184 (0.381–3.683)	0.770
Lymphocytes (> 3.2 vs. ≤ 3.2)	2.289 (0.694–7.549)	0.174	-	-
Monocytes (> 0.6 vs. ≤ 0.6)	3.298 (1.467–7.417)	**0.004**	1.137 (0.387–3.340)	0.815
Platelets (> 300 vs. ≤ 300)	1.488 (0.608–3.642)	0.384	-	-
SII (> 655.54 vs. ≤ 655.54)	1.925 (0.939–3.947)	0.074	-	-
SIRI (> 1.12 vs. ≤ 1.12)	3.335 (1.626–6.841)	**0.001**	3.205 (1.285–7.997)	**0.013**
**OS**				
Gender (female vs. male)	0.336 (0.078–1.454)	0.145	-	-
Age (> 55 vs. ≤ 55)	3.599 (1.447–8.955)	**0.006**	4.608 (1.694–12.536)	**0.003**
Smoke (yes vs. no)	1.321 (0.537–3.254)	0.544	-	-
AJCC stage (8th) (III-IVb vs. I-II)	4.322 (0.998–18.718)	0.051	-	-
Tumor classification (T_3-4_ vs. T_1-2_)	1.118 (0.454–2.751)	0.809	-	-
Node classification (N_2-3_ vs. N_0-1_)	1.963 (0.772–4.992)	0.157	-	-
Metastasis (Yes vs. No)	5.139 (0.684–38.608)	0.112	-	-
WHO pathologic type (TypeIIb vs. TypeIIa vs. TypeI)	0.556 (0.209–1.481)	0.240	-	-
Leukocytes (> 9.5 vs. ≤ 9.5)	5.690 (2.155–15.019)	** < 0.001**	3.797 (0.879–16.397)	0.074
Neutrophils (> 6.3 vs. ≤ 6.3)	3.366 (1.211–9.360)	**0.020**	0.513 (0.129–2.037)	0.343
Lymphocytes (> 3.2 vs. ≤ 3.2)	2.706 (0.624–11.728)	0.183	-	-
Monocytes (> 0.6 vs. ≤ 0.6)	5.550 (2.181–14.125)	** < 0.001**	1.425 (0.407–4.983)	0.580
Platelets (> 300 vs. ≤ 300)	1.076 (0.313–3.692)	0.908	-	-
SII (> 655.54 vs. ≤ 655.54)	1.550 (0.623–3.855)	0.346	-	-
SIRI (> 1.12 vs. ≤ 1.12)	4.793 (1.885–12.186)	**0.001**	5.730 (1.831–17.926)	**0.003**

*EBV DNA* Epstein-Barr virus DNA, *AJCC* American Joint Committee on Cancer, *WHO* World Health Organization, *SII* Systemic immune-inflammation index, *SIRI* Systemic inflammation response index

In the multivariate Cox regression model, only SIRI were associated with PFS and OS in training cohort (all *p* < 0.05) (Table 2). In validation cohort, the age and SIRI were associated with PFS and OS (all *p* < 0.05) (Table 3). Only SIRI remained an independent prognostic factor in both training cohort and validation cohort.

### ROC curves analysis

**Fig. 4:**
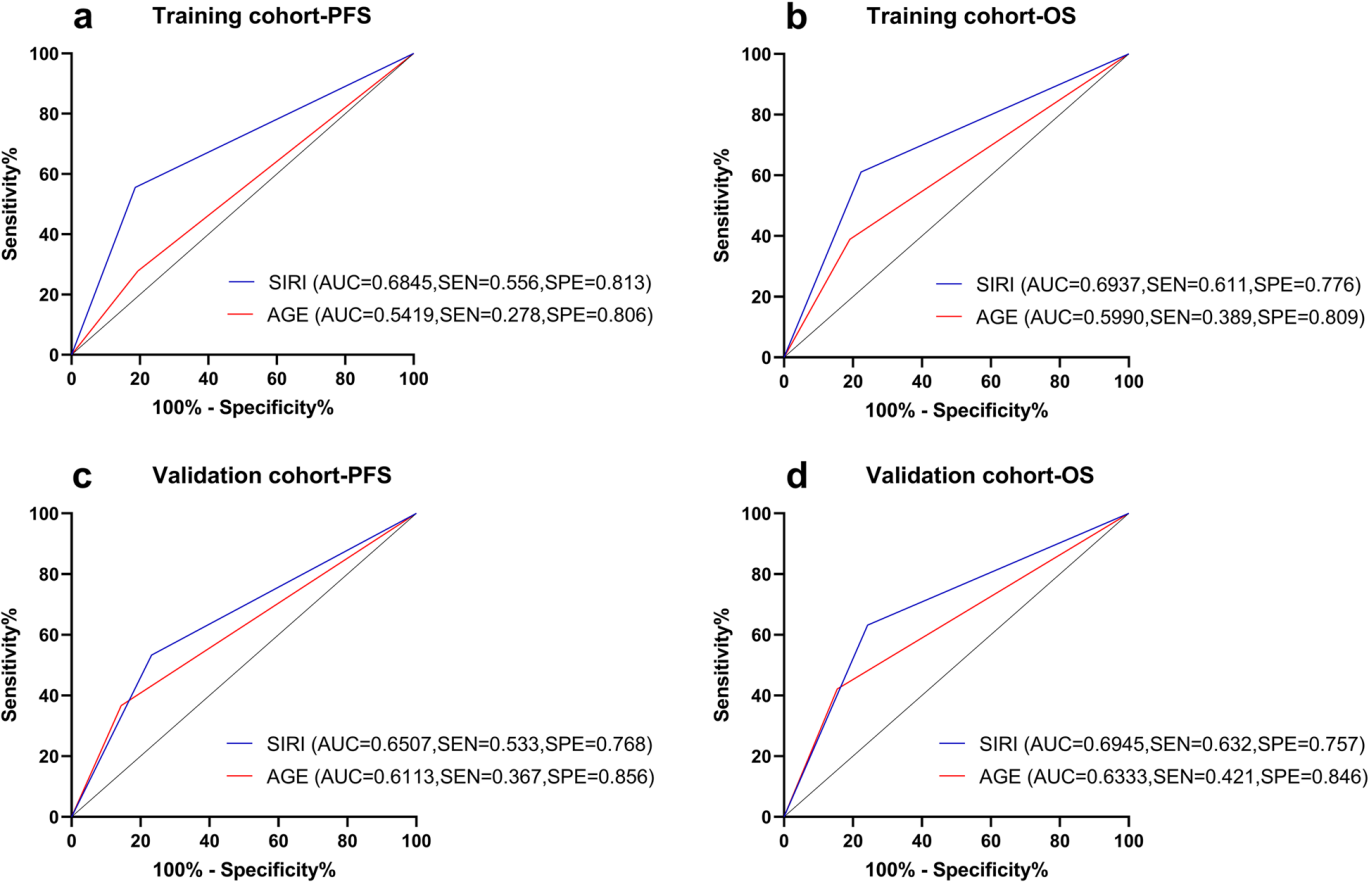
ROC curves analysis for comparing the prognostic potential of independent prognostic factors. **a** Prediction of PFS in training cohort; **b** Prediction of OS in training cohort; **c** Prediction of PFS in validation cohort; **d** Prediction of OS in validation cohort

Since in the multivariate analysis of validation cohort, both age and SIRI could be considered as independent prognostic factors in NPC patients with negative EBV DNA, we included age and SIRI in the ROC curves to compare their prognostic value. The results demonstrated that the area under the curve (AUC) values of SIRI larger than the AUC values of age for PFS and OS in validation cohort. Although the age was not an independent prognostic for NPC patients with negative EBV DNA in training cohort, we finally included age and SIRI for the ROC analysis in training cohort, and the final results were consistent with the validation cohort (Fig. 4).

### Relationship between clinical characteristics and SIRI

The SIRI levels were significantly correlated with AJCC stage and tumor classification in training cohort (all *p* < 0.05). However, these results were not confirmed in validation cohort. The SIRI levels were significantly correlated with gender and smoke in validation cohort (all *p* < 0.05) (Table 4).

**Table 4:** Relationship between clinical characteristics and SIRI of NPC patients with negative EBV DNA in training and validation cohorts

Variables	Training cohort (*n* = 170)			Validation cohort (*n* = 155)		
	SIRI		*p*	SIRI		*p*
	≤ 1.12	> 1.12		≤ 1.12	> 1.12	
Gender, No.(%)			0.620			**0.038**
male	81 (64.8)	31 (68.9)		78 (70.9)	39 (86.7)	
female	44 (35.2)	14 (31.3)		32 (29.1)	6 (13.3)	
Age, No.(%)			0.293			0.520
≤ 55	101 (80.8)	33 (73.3)		88 (80.0)	38 (84.4)	
> 55	24 (19.2)	12 (26.7)		22 (20.0)	7 (15.6)	
Smoke			0.974			**0.037**
no	83 (66.4)	30 (66.7)		69 (62.7)	20 (44.4)	
yes	42 (33.6)	15 (33.3)		41 (37.3)	25 (55.6)	
AJCC stage (8th), No.(%)			**0.037**			0.059
I-II	43 (34.4)	8 (17.8)		39 (35.5)	9 (20.0)	
III-IVb	82 (65.6)	37 (82.2)		71 (64.5)	36 (80.0)	
Tumor classification, No.(%)			**0.010**			0.622
T1-T2	61 (48.8)	12 (26.7)		61 (55.5)	23 (51.1)	
T3-T4	64 (51.2)	33 (73.3)		49 (44.5)	22 (48.9)	
Node classification, No.(%)			0.113			0.081
N0-N1	70 (56.0)	19 (42.2)		61 (55.5)	18 (40.0)	
N2-N3	55 (44.0)	26 (57.8)		49 (44.5)	27 (60.0)	
Metastasis, No.(%)			0.656			0.498
Non-metastasis	121 (96.8)	43 (95.6)		109 (99.1)	44 (97.8)	
Metastasis	4 (3.2)	2 (4.4)		1 (0.9)	1 (2.2)	

*EBV DNA* Epstein-Barr virus DNA, *AJCC* American Joint Committee on Cancer, *SIRI* Systemic inflammation response index

### Relationship between EBV DNA status and inflammatory parameters or clinical characteristics in all 795 NPC patients

To further confirm that SIRI could be used as a good prognostic indicator for NPC patients with negative EBV DNA, we analyzed the correlation between EBV DNA status and inflammatory parameters. In the overall population (*n* = 795), EBV DNA status was not associated with any inflammatory parameters (Supplement Table 1). However, EBV DNA status was significantly associated with AJCC stgae (*p* < 0.001), Tumor classification (*p* < 0.001) and Node classification (*p* < 0.001), the NPC patients with positive EBV DNA had more advanced stages (Supplement Table 2).

## Discussion

Multiple reports have shown that pretreatment negative EBV DNA patients have better survival and prognosis than patients with high plasma EBV DNA levels [7, 13, 14]. However, many patients with negative EBV DNA still have disease progression, recurrence and metastasis, and the prognostic factors of these patients have rarely been studied. Our findings demonstrated that the SIRI had prognostic value in NPC patients with negative EBV DNA and that high SIRI was associated with worse PFS and OS in these patients.

In this study, we found that high SIRI level was closely related to worse 5-year PFS and OS both in in training cohort and validation cohort, these findings were consistent with previous studies [19, 20]. Currently, inflammation is considered a hallmark of cancer and has the ability to activate invasion and metastasis, induce angiogenesis, and sustain proliferative signaling by many scholars [16, 23]. But, there is still no accurate explanation for the mechanism related to the prognosis of SIRI in cancer patients. For now, the prognostic role of SIRI can be explained by the function of its own components. The neutrophils can release a variety of cytokines, such as interleukin-10 (IL-10) and transforming growth factor-β (TGF-β), to inhibit T cell proliferation and activation [24]. Lymphocytes are often able to inhibit tumor proliferation and metastasis, and kill tumor cells through their immune function (secrete interferon gamma (INF-γ) and tumor necrosis factor (TNF-α)) [25]. Monocytes and monocyte-derived M2 macrophages play important roles in tumor growth, invasion and suppression of antitumor immunity and dissemination [26]. In addition, the number of monocytes and tumor burden are often closely related [27]. However, there was no prognostic value of SII in NPC patients with negative EBV DNA. The reason for this result may be due to the different functions between platelets and monocytes in cancer. Platelets are closely related to cancer metastasis, and the mechanism for this phenomenon is that platelets support cancer cell migration and metastasis formation by stimulating and aiding cancer cell adhesion and extravasation [28]. However, patients with negative EBV DNA have far fewer distant metastases than patients with positive EBV DNA, so the SII could not achieve a good predictive effect in negative EBV DNA patients. The function of monocytes not only promotes distant metastasis of cancer cells, but also plays an important role in inducing tumor resistance, mediating immune escape, and promoting neovascularization [29]. Therefore, this also explains why only SIRI has prognostic value in NPC patients with negative EBV DNA.

To further confirm inflammatory parameters could be considered as possible prognostic factors for NPC patients with negative EBV DNA, we conducted a correlation analysis for EBV DNA status and clinical characteristics or inflammatory parameters in all patients. We found that NPC patients with positive EBV DNA had advanced AJCC stage, Tumor classification and Node classification. This conclusion was consistent with Leung et al. [10] and Peng et al. [12], who found that EBV DNA levels were related to AJCC stage. In addition, EBV DNA load was significantly correlated with both circulating tumor cells (CTCs) and tumor burden [11, 30], which explained why NPC patients with positive EBV DNA had a worse prognosis. But in our study, EBV DNA status was not related to peripheral blood inflammatory cells and inflammatory parameters, which meant that EBV infection did not affect the change in systemic inflammation. Current studies have shown that peripheral plasma EBV DNA comes from tumor cells, and EBV infection usually changes local inflammation around the tumor tissue [31], but the systemic inflammatory changes in EBV-infected patients are not obvious. Therefore, we considered that SIRI was a prognostic factor not affected by EBV infection and had good prognostic value for NPC patients with negative EBV DNA, so it was more suitable as a prognostic factor for these patients.

Due to the SIRI was an independent prognostic factor in NPC patients with negative EBV DNA, we analyzed the correlation between SIRI and other clinical characteristics. The SIRI levels were correlated with AJCC stage and tumor classification in training cohort. However, these results were not confirmed in validation cohort. Therefore, the conclusions of this part still need a large sample of data for further verification.

Since both age and SIRI were independent prognostic factors for NPC patients with negative EBV DNA in validation cohort, we further evaluated the predictive value of age and SIRI by ROC curves. We found that the prognostic value of SIRI was greater than that of age in both training cohort and validation cohort. This confirmed that the SIRI in NPC patients with negative EBV DNA could serve as a strong independent prognostic factor. In previous study, scholars have always combined the pretreatment plasma EBV DNA with clinical features or other biomarkers such as age, sex, T classification, N classification, body mass index (BMI), lactate dehydrogenase (LDH) and SII, to provide more accurate prognostic prediction for patients with NPC [1, 32, 33]. However, the prognosis and survival of NPC patients with negative EBV DNA were often ignored. To the best of our knowledge, this is the first study to investigate inflammatory immune indicators in NPC patients with negative EBV DNA for survival. In the past, scholars have always focused on the survival and prognosis of NPC patients with positive EBV DNA. Patients with negative EBV DNA and high SIRI level should be given more attention, necessary pretreatment inspections and close follow-up monitoring, as well as the formulation of more precise treatment strategies according to the specific situation.

However, our study has some limitations. First, this was a retrospective study conducted in a single institution, and potential bias could not be avoided. Second, Due to limited data on EBV DNA during or after treatment, a study of changes in EBV DNA and SIRI was not performed. Therefore, more large-scale prospective, multi-center, randomized clinical trials are still needed for further verification and research.

## Conclusion

In summary, the results of our study demonstrated that the SIRI was a significant predictor for prognosis in NPC patients with negative EBV DNA. In addition, inflammatory parameters did not change with EBV DNA status. Therefore, more precise and individualized treatment management strategies can be provided for NPC patients with negative EBV DNA and high SIRI level.

## Supplementary Information

**Additional file 1: Supplementary file 1.  **The detection method for EBV DNA.

**Additional file 2: SupplementTable 1.** Univariate logisticregression analyses of inflammatory parameters as a function of EBV DNA(negative, positive) for all NPC.

**Additional file 3: Supplement Table 2.** Relationship between clinical characteristicsand EBV DNA status of all NPC patients (*n*=795).

## Data Availability

The data used to support the findings of this study are available from the corresponding author upon request.

## Abbreviations

AC: Adjuvant Chemotherapy
AJCC: American Joint Committee On Cancer
AUC: Area Under The Curve
BMI: Body Mass Index
CCRT: Concurrent Chemoradiotherapy
CTCs: Circulation Tumor Cells
CTV: Clinical Target Volume
EBV: Epstein–Barr Virus
ECT: Emission Computed Tomography
GTVnd: Positive Neck Lymph Node Area
GTVnx: Gross Tumor Volume Of The Nasopharynx
IC: Induction Chemotherapy
IMRT: Intensity-Modulated Radiation Therapy
LDH: Lactate Dehydrogenase
MRI: Magnetic Resonance Imaging
NCCN: National Comprehensive Cancer Network
NPC: Nasopharyngeal Carcinoma
OR: Odds Ratio
OS: Overall Survival
PET-CT: Positron Emission Tomography-Computed Tomography
PFS: Progression-Free Survival
SII: Systemic Immune-inflammation Index
SIRI: Systemic Inflammation Response Index
TTP: Time To Progression
