# Supplementary material for: Prognostic value of systemic inflammation response index in nasopharyngeal carcinoma with negative Epstein-Barr virus DNA
Source: BMC Cancer. 2022 Aug 5;22:858. doi: 10.1186/s12885-022-09942-1 (PMC9356473; doi:10.1186/s12885-022-09942-1)
Supplement: Supplementary file 1 — Additional file 1: Supplementary file 1. The detection method for EBV DNA. [file 12885_2022_9942_MOESM1_ESM.doc]

**Supplementary file 1** The detection method for EBV-DNA

Plasma EBV DNA measurements were completed in the Laboratory Medicine Center of Nanfang Hospital, Southern Medical University. Venous blood samples (5 ml/each case) were collected before treatment and put in ethylenediaminetetraacetic acid (EDTA) tubes. The collected blood was then centrifuged at 1500×g for 5 min at 4°C. Plasma total DNA was extracted using the QIAamp blood kit (Qiagen, Hilden, Germany). The EBV genome was amplified by real-time quantitative PCR (RT-qPCR) using an EBV RT-qPCR kit and primers 5′-GCTGCGCTGCTGCTATCTT-3′ (forward) and 5′-CAAGCCCACTCCCCTGTCT-3′ (reverse) according to the manufacturer’s instructions (Liferiver, Shanghai, China). The GAPDH gene was amplified as a control using the primers 5′-GGCGACGCAAAAGAAGATG-3′ (forward) and 5′-CCGTTGACTCCGACCTTCAC-3′ (reverse). PCR conditions were as follows: initial denaturation at 95°C for 10 minutes, followed by 40 cycles of denaturation at 95°C for 15 seconds and amplification at 56°C for 1 minute.
