# Supplementary material for: Prognostic value of systemic inflammation response index in nasopharyngeal carcinoma with negative Epstein-Barr virus DNA
Source: BMC Cancer. 2022 Aug 5;22:858. doi: 10.1186/s12885-022-09942-1 (PMC9356473; doi:10.1186/s12885-022-09942-1)
Supplement: Supplementary file 2 — Additional file 2: SupplementTable 1. Univariate logisticregression analyses of inflammatory parameters as a function of EBV DNA(negative, positive) for all NPC. [file 12885_2022_9942_MOESM2_ESM.doc]

| **Supplement Table 1.** Univariate logistic regression analyses of inflammatory parameters as a function of EBV DNA (negative, positive) for all NPC patients (n=795) | | |
| --- | --- | --- |
| Variables | Univariate analysis | |
|  | OR (95%CI) | *p* |
| Leukocytes | 1.025 (0.955-1.101) | 0.490 |
| Neutrophils | 1.044 (0.961-1.135) | 0.309 |
| Lymphocytes | 0.972 (0.798-1.185) | 0.781 |
| Monocytes | 1.852 (0.778-4.407) | 0.163 |
| Platelets | 1.000 (0.998-1.003) | 0.674 |
| SII | 1.000 (1.000-1.001) | 0.260 |
| SIRI | 1.083 (0.947-1.238) | 0.243 |
| SII: Systemic immune-inflammation index; SIRI, Systemic inflammation response index | | |
