# Supplementary material for: Prognostic value of systemic inflammation response index in nasopharyngeal carcinoma with negative Epstein-Barr virus DNA
Source: BMC Cancer. 2022 Aug 5;22:858. doi: 10.1186/s12885-022-09942-1 (PMC9356473; doi:10.1186/s12885-022-09942-1)
Supplement: Supplementary file 3 — Additional file 3: Supplement Table 2. Relationship between clinical characteristicsand EBV DNA status of all NPC patients (n=795). [file 12885_2022_9942_MOESM3_ESM.doc]

| **Supplement Table 2.** Relationship between clinical characteristics and EBV DNA status of all NPC patients (n=795) | | | |
| --- | --- | --- | --- |
| Variables | EBV DNA status | | |
|  | Negative | Positive | *p* |
| Gender, No.(%) |  |  | 0.363 |
| male | 229 (70.5) | 345 (73.4) |  |
| female | 96 (29.5) | 125 (26.6) |  |
| Age, No.(%) |  |  | 0.139 |
| ≤ 55 | 260 (80.0) | 355 (75.5) |  |
| > 55 | 65 (20.0) | 115 (24.5) |  |
| Smoke |  |  | 0.303 |
| no | 202 (62.2) | 275 (58.5) |  |
| yes | 123 (37.8) | 195 (41.5) |  |
| AJCC stage (8th), No.(%) |  |  | **<0.001** |
| Ⅰ-Ⅱ | 99 (30.5) | 62 (13.2) |  |
| Ⅲ-Ⅳb | 226 (69.5) | 408 (86.8) |  |
| Tumor classification, No.(%) |  |  | **<0.001** |
| T1-T2 | 157 (48.3) | 161 (34.3) |  |
| T3-T4 | 168 (51.7) | 309 (65.7) |  |
| Node classification, No.(%) |  |  | **<0.001** |
| N0-N1 | 168 (51.7) | 172 (36.6) |  |
| N2-N3 | 157 (48.3) | 298 (63.4) |  |
| Metastasis, No.(%) |  |  | 0.082 |
| Non-metastasis | 317 (97.5) | 447 (95.1) |  |
| Metastasis | 8 (2.5) | 23 (4.9) |  |
| EBV DNA: Epstein-Barr virus DNA; AJCC: American Joint Committee on Cancer | | | |
